# Distribution characteristics and assessment of the content of heavy metals in small rivers of the Ulba riv. basin in the mining regions of East Kazakhstan

**DOI:** 10.1039/d5ra00801h

**Published:** 2025-04-08

**Authors:** Madina Dyussembayeva, Azhar Tashekova, Yerbol Shakenov, Vladimir Kolbin, Nazgul Nurgaisinova, Ainur Mamyrbayeva, Marija Abisheva

**Affiliations:** a The Institute of Radiation Safety and Ecology of the National Nuclear Center of the Republic of Kazakhstan 2 Beibyt Atom St., Kurchatov City 180010 Republic of Kazakhstan esenzholova@nnc.kz

## Abstract

Water quality of small rivers in the Ulba basin has been assessed in the impact zone of the mining industry of the Ridder region in East Kazakhstan. Sixteen elements in the waters of small rivers and general chemical water indices were determined using mass spectrometry. The waters of the small rivers under investigation were primarily ultra-fresh and slightly alkaline. The chemical composition of the examined waters was characterised as a sodium–potassium sulphate type, a calcium–magnesium bicarbonate type, and a mixed chemical type, namely, sodium–calcium bicarbonate–sulphate. These waters do not conform to the Health Standards established by the Republic of Kazakhstan, as indicated by the hardness indices for the Filippovka and Bystrukha riv. The cadmium content exceeded the MPC set by the Health Standards of the Republic of Kazakhstan in the waters of the Ulba riv. (up to 21 MPC), Tikhaya (up to 5 MPC) and Filippovka riv. (up to 3 MPC) in 65%, 88% and 18% of water samples, respectively. Single samples were also found to contain elevated concentrations of manganese (Filippovka riv. and Breksa riv.) and ferrum (Breksa riv.). According to the standards set by the World Health Organization (WHO) and the US MPC, exceedances of manganese, aluminium, iron, and cadmium contents in the waters of the Ulba, Filippovka, Breksa, and Bystrukha rivers were observed, ranging from 1 to 7 times. The highest exceedances were recorded in the waters of the Ulba river, with manganese concentrations exceeding the WHO standards by 4 times and US EPA standards by 6.4 times and cadmium concentrations exceeding the WHO standards by 7 times and US EPA standards by 4.2 times. In most water samples from Tikhaya and Ulba riverbeds and in the upper reach of the Filippovka riv, high and average levels of water contamination were revealed (according to the pollution index of heavy metals (HPI)). Alternatively, low contamination levels (<15) with no elevated concentrations of heavy metals were observed in the waters of Zhuravlikha, Malaya Zhuravlikha, Gromotukha, Khariuzovka, Bystrukha and Breksa.

## Abstract 2

Water quality of small rivers in the Ulba basin has been assessed in the impact zone of the mining industry of the Ridder region in East Kazakhstan.

## Graphical abstract

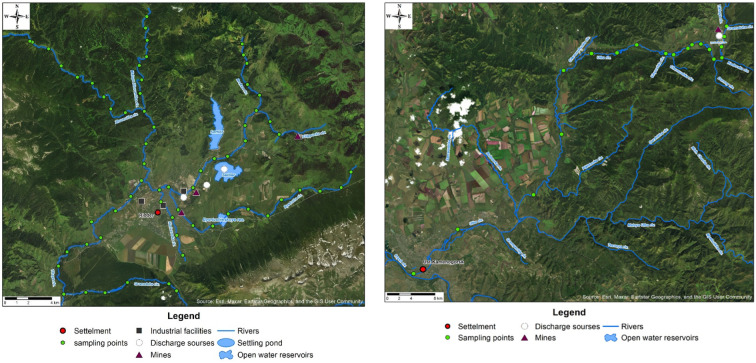

## 1 Introduction

In the modern world, ecological impact assessment of mining facilities on aquatic ecosystems is highly relevant.^1–3^ Most research undertaken in Kazakhstan aims to study anthropogenic stress and biodiversity challenges related to large and transboundary rivers,^4–7^ with a particular focus on the Irtysh riv., which is the major waterway in the region.^8–11^

Small rivers, much like the upper reaches in larger landscape systems, serve as indicators of the ecological condition of the regional areas and natural zones. This is because they are the first to be impacted by the consequences of adverse effects stemming from economic activities. The ecosystems of low water rivers with a low diluting capacity are marked by poor resistance to anthropogenic effects. The most significant transformation in the chemical composition and water quality of small rivers in the Ulba riv. basin is attributed to the man-made impacts caused by mining activities. Previous investigations into trace elements in this region^12,13^ have a limited scope, are highly specialised, and are fragmented, focusing on a narrow range of elemental constituents. No extensive research has been conducted on the elemental constituents of the major small rivers in the mining regions in East Kazakhstan, particularly in the vicinity of the Ridder c. Hence, research into their current ecological conditions is highly relevant.

Owing to the long-term polymetallic and gold ore mining in this region, water resource contamination with heavy metals poses a real threat to the aquatic medium and human health because of the toxicity, tolerance and bioaccumulation of these contaminants.^14–17^ According to the annual information materials provided by Kazgidromet,^18,19^ the small rivers of the Ridder c. such as Breksa, Tikhaya and Ulba are highly contaminated water streams.

This study aims to study spatial variations in the concentrations of heavy metals in the small rivers of the Ulba basin in East Kazakhstan and evaluate the water quality in the impact zone of the mining industry in the region.

## 2 Materials and research techniques

### 2.1 Scope of research

The Ridder area of the East Kazakhstan region is notable for a well-developed hydrological network represented by the Breksa, Filippovka, Bystrukha, Khariuzovka, Malaya Zhuravlikha, Zhuravlikha, Tikhaya, Gromotukha and Ulba riv. These are predominantly mountain rivers, whose heads are produced by melting snow and the glacier cover of the Altai mountain range with spring turbulent floods and extended high waters.^20^ It should be noted that the head of the Tikhaya riv. is produced within the Ridder c. after the confluence of the Zhuravlikha, Filippovka, Bystrukha, Khariuzovka and Malaya Zhuravlikha mountain rivers, and after entering the Gromotukha riv., it forms the Ulba riv. The Ulba, in turn, is one of the large right-bank tributaries of the transboundary Irtysh riv.

The region of interest is the Rudny Altai province, particularly the Ridder (Leninogorsk) ore district. There are many pyrite–copper–zinc, barite–pyrite–polymetallic and pyrite–polymetallic deposits in this region (the main ones are Tishinskoye, Ridder-Sokolnoye, Shubinskoye and Dolinnoye deposits), which are volcanogenic formations mainly of the Devonian age. The main elements of industrial importance are Zn, Cu and Pb and associated ones including Ba, Au, Ag, Cd, Sb, As, Bi, Sn, Se, Te, Hg, Ga, In, Ge, Tl, and Co.^21^

The industrial sites of facilities and, consequently, their tailing dumps, sludges, ore and overburden dumps are located within the city of Ridder and near the rivers of interest. For example, one of the largest facilities in the region is the ridder mining and processing complex, which produces and reprocesses polymetallic ores from deposits located near the city of Ridder in the East Kazakhstan region. The operating facilities of the ridder mining and processing complex include the Ridder-Sokolny, Tishinsky and Dolinny underground mines, a dressing plant and other auxiliary subdivisions. The primary types of industrial output products are copper, lead-zinc, gold ores and their concentrates as well as unprocessed lead and unprocessed zinc.^22^

Over the years of the operation of the mining complex, treated effluents from the Ridder facilities have been discharged into the Filippovka, Bystrukha, Khariuzovka and Ulba riv.^23^ There are also known cases of the off-normal discharges of industrial waters into the small rivers of the Ridder c. by large mining facilities in the region.^24^

It should be noted that the water intake of the ridder water utility is located on the Gromotukha mountain river, which is the major source of water supply for the local population. The head of the Gromotukha riv. is in the high-mountain part of the Ivanovsky ridge (Rudny Altai) outside the anthropogenic impact zone. Nevertheless, the supervision of the water quality and resource potential of this river is a highly relevant issue.

### 2.2 Sampling

**Fig. 1:**
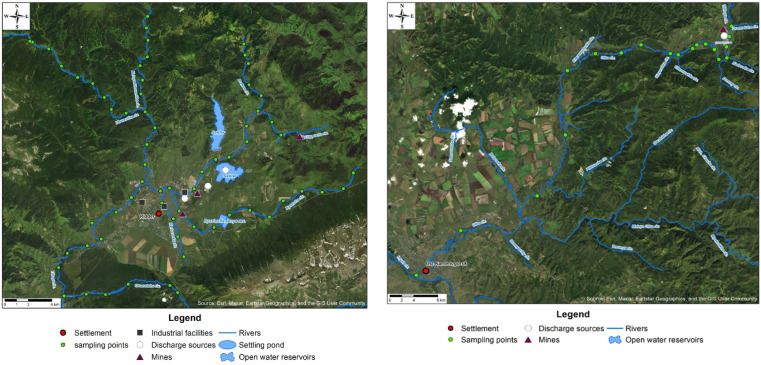
Sampling points on the small rivers of the Ulba riv. basin. The map depicts discharge points of effluents according to the data.^25^

Water samples were collected during the summer low-water period, from July 11 to July 22, 2023, because pollutants are significantly diluted and washed away from large catchment areas during the spring and autumn flood seasons. The locations of sampling points were identified using GPS. River water samples were uniformly collected along the length of water streams predominantly with a sampling spacing of 2 km and at the Ulba riv. with a spacing of 2 km, 5 km and 15 km covering the upper reaches of rivers, middle sections and estuaries, respectively (Fig. 1). Altogether, 85 sites were surveyed. To assess the potential impact of the identified contamination sources, such as the discharges of mine waters and effluents, samples were uniformly collected along the entire riverbeds at points above and below discharges (Fig. 1).

Natural waters were sampled as per the state standard GOST 31861-2012.^26^ River water was sampled from the subsurface, that is, 15 cm below the water surface.

The following operations were carried out when sampling of water was done for elemental analysis: water filtration using a 0.45 μm filter to remove mechanical impurities and sample preservation by adding 3 ml of ACS-grade concentrated nitric acid (HNO_3_) per 1 l of a water sample.

Filtration and preservation were accomplished *in situ*. For the general chemical analysis of water (chlorides, sulphates, total hardness, and total salinity), 1.5 l water samples were collected without preserving them in nitric acid.

### 2.3 Water sample analyses of chemical elements and chemical and physical parameters

The concentration of chemical elements was determined using mass spectrometry and inductively coupled plasma atomic emission spectrometry (ICP-AES). Analyses were conducted using an Agilent 7700 quadrupole mass spectrometer from Agilent Technologies and an iCAP 6300 Duo inductively coupled plasma atomic emission spectrometer from Thermo Scientific. Using these techniques, the contents of 16 elements, namely, Li, Al, V, Mn, Fe, Co, Ni, Cu, Zn, As, Sr, Mo, Cd, Ba, Pb, and U, were determined, with detection limits of 0.01–100 μg l^−1^ and an uncertainty of 10–15%. Measurements were performed in triplicate. It should be noted that ferrum was mainly determined using ICP-AES.

To calibrate spectrometers, the calibration solutions of analytes were used, namely, 10 μg l^−1^ and 20 μg l^−1^ for ICP-MS and 1000 μg l^−1^ and 5000 μg l^−1^ for ICP-AES. To plot calibration graphs, multi-element reference standard solutions were used containing metals manufactured by PerkinElmer (USA; No. 9300231, No. 9300233, and No. 9300235), with a rated certified value of metal content of 10 mg l^−1^ and an uncertainty of the certified value of 0.5% (dilution factor *k* = 2).

For elemental analysis, undiluted water samples were used for the ICP-AES method and pre-diluted samples (diluted no more than five times with 1% nitric acid) were used for the ICP-MS method.

Measurement quality was overseen by measuring a calibration solution every 10 samples. To control the accuracy of measurements, a calibration standard solution of the metal composition from Inorganic Ventures IV-ICP-MS-71A, CMS-1 (Inorganic Ventures, USA) was used. If the calibration result was unsatisfactory (deviation of a calibration graph was 8–10%), the instrument was recalibrated using new background parameters.

The analysis was carried out as per the procedure reported in the GOST ISO 17294-2-2019 ‘Application of inductively coupled plasma mass spectrometry. Part 2: determination of certain elements including uranium isotopes’.^27^

To determine the chemical and physical parameters and contents of macrocomponents in water, a general chemical analysis of the water composition was carried out using standard procedures,^28^ which included the analysis of the pH level, salinity, hardness rate, and macrocomponents of the main composition (Na^+^, K^+^, Ca^2+^, Mg^2+^, Cl^−^, HCO_3_^−^, and SO_4_^2−^).

### 2.4 Quality control

Water samples were analysed by accredited laboratories (ISO 17025:2009) at the Institute of Radiation Safety and Ecology in the Republican State Enterprise National Nuclear Center of the Republic of Kazakhstan, Kurchatov t. Research was undertaken using analytical and testing equipment calibrated and tested in accordance with the Law of the Republic of Kazakhstan dated June 7, 2000 No. 53-II ‘On the assurance of uniformity of measurements’.

All chemical reagents and reactants were of analytical grade quality. Before use, all glass and plastic containers were soaked in a 14% HNO_3_ solution for 24 hours and rinsed with distilled water. The quality control of measurements was performed by analysing the calibration solution every 10 samples. To verify the accuracy (confirmation) of calibration characteristics, a standard solution of a metal composition (Inorganic Ventures IV-ICP-MS-71A, CMS-1 (Inorganic Ventures, USA)) was used for sample preparation.

### 2.5 Processing of results

The index of heavy metal pollution (HPI) is an assessment technique that demonstrates the aggregated impact of individual heavy metals on the total water quality. The evaluation system includes arbitrary values from 0 to 1, which are chosen depending on the importance of individual quality indices, or the HPI can be estimated by comparing values with recommendations for the relevant parameters.^29–32^ The HPI is based on the technique of a weighted arithmetic mean, that is, weighted values are used to establish a rating scale for each selected parameter; then, a contamination parameter index is chosen.^33^

To calculate Wi, the Kazakhstani standards of potable water were used for the elements of interest,^34^ except uranium, for which a standard is not provided. Instead, uranium standards established by the WHO were used for calculations.^35^

The model formula for the HPI^32^ is as follows:
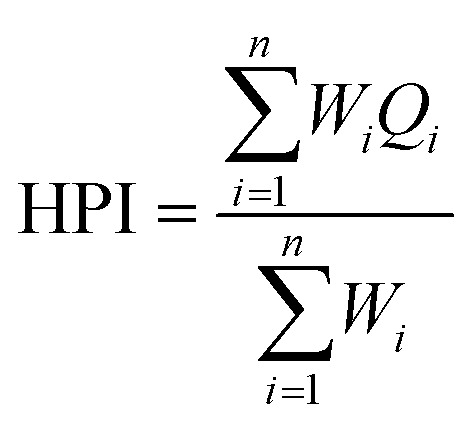
where *Q*_*i*_ is a subindex of the *i*th parameter, *W*_*i*_ is the specific gravity of the *i*th parameter, and *n* is the number of parameters in question.

The formula used to compute *Q*_*i*_ is as follows:
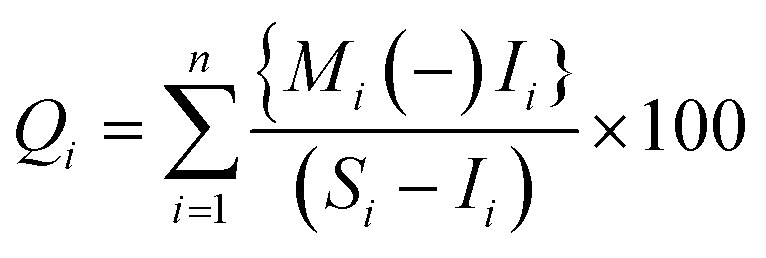
where *M*_*i*_ is the controlled content of heavy metals for the *i*th parameter, *I*_*i*_ is the ideal value of the *i*th parameter, and *S*_*i*_ is the standard value of the *i*th parameter. The sign (−) indicates the numerical difference between two values while ignoring the algebraic sign. The critical contamination index of drinking water obtained by Prasad^31^ is 100. However, Edet and Offiong^36^ used a modified scale of three classes. The classes were divided into low, medium and high using HPI values of 15, 15–30 and 30, respectively.

## 3 Results and discussion

### 3.1 Macrocomponent composition of the small rivers of the Ulba riv. basin

The chemical compositions of the waters of small rivers are presented in Table 1.

**Table 1:** Chemical composition of the small rivers of the Ulba riv. basin^a^

River name	pH	Salinity, mg l^−1^	Hardness, mmol l^−1^	Content of cations, mg l^−1^			Content of anions, mg l^−1^		
				Na^+^ + K^+^	Ca^2+^	Mg^2+^	Cl^−^	HCO_3_^−^	SO_4_^2−^
Zhuravlikha riv. (*n* = 6)	**7.9**	**68**	**0.5**	**20**	**5**	**1.0**	**2.0**	**20**	**31**
	*7.6–8.3*	*45–80*	*0.5–1*	*15–25*	*1–10*	*1.0–2.5*	*2.0–3.5*	*15–50*	*20–40*
	3%	19%	35%	20%	55%	52%	27%	50%	21%
Malaya Zhuravlikha riv. (*n* = 3)	**8.1**	**110**	**1.0**	**25**	**7.0**	**2.0**	**2.0**	**50**	**45**
	*7.8–8.5*	*90–120*	*0.5–1.0*	*20–60*	*1.0–10*	*2.0–5.0*	*2.0–3.0*	*40–50*	*40–55*
	4%	14%	35%	62%	76%	58%	25%	12%	16%
Breksa riv. (*n* = 2)	**8.6**	**118**	**1.7**	**12**	**22**	**6.0**	**2.5**	**86**	**31**
	*8.4–8.7*	*115–120*	*1.5–1.8*	*8–16*	*19–25*	*6.0–6.1*	*2.0–3.0*	*79–92*	*25–37*
	2%	3%	13%	47%	19%	1.2%	28%	11%	27%
Filippovka riv. (*n* = 6)	**8.6**	**168**	**30**	**5**	**38**	**13**	**5**	**96**	**80**
	*7.7–8.8*	*95–390*	*–—*	*0.5–50*	*5–95*	*1–20*	*1.0–8.5*	*30–165*	*25–195*
	5%	56%	0%	159%	75%	55%	86%	46%	67%
Bystrukha riv. (*n* = 5)	**8.1**	**60**	**30**	**15**	**5**	**5**	**2.0**	**25**	**25**
	*7.8–8.5*	*55–115*	*25–30*	*15–60*	*5–10*	*0.5–5*	*2.0–3.0*	*25–30*	*20–30*
	4%	36%	8%	79%	37%	71%	20%	10%	16%
Khariuzovka riv. (*n* = 2)	**8.0**	**53**	**0.2**	**14**	**4.5**	<0.5	**2.5**	**18**	**23**
	*7.9–8.0*	*40–65*	*–—*	*11–16*	*4.0–5.0*		*–*	*15–20*	*15–30*
	1%	34%	0%	26%	116%		0%	20%	47%
Gromotukha riv. (*n* = 2)	**7.8**	**65**	**0.5**	**20**	**5**	**1.0**	**2.3**	**17.5**	**35**
	*7.7–7.8*	*60–70*	*–*	*–*	*–*	*–*	*2.0–2.5*	*15–20*	*30–40*
	1%	11%	0%	0%	0%	0%	16%	20%	20%
Tikhaya riv. (*n* = 4)	**8.2**	**105**	**1.0**	**21**	**15**	**4.0**	**2.3**	**46**	**45**
	*8.0–8.6*	*55–130*	*–*	*0.5–30*	*10–20*	*2.0–5.0*	*2.0–3.5*	*32–55*	*10–70*
	3%	33%	0%	69%	27%	40%	28%	23%	59%
Ulba riv. (*n* = 9)	**8.1**	**100**	**1.0**	**15**	**15**	**2.5**	**3.5**	**45**	**40**
	*8.0–8.7*	*80–130*	*1.0–2.0*	*10–22*	*10–30*	*1.5–6.0*	*2.0–8.5*	*35–95*	*40–50*
	3%	15%	30%	22%	37%	45%	53%	36%	8%
MPC of water^34^	6–9	1000	7	—	—	—	—	350	500

a Bold–median, italic–min–max, Cv in brackets, %-variation coefficient.

The acid–alkaline conditions of the waters of the studied rivers are characterized by a change in the pH of waters from 7.6 to 8.8 and, in the most cases, undergo a slightly alkaline reaction. In the waters of the Breksa riv. and Filippovka riv., the pH increases significantly from 8.5 to 8.8, and waters become alkaline.

The waters of the small rivers studied are mainly ultra-fresh with a mineralization from 40 to 180 mg l^−1^. Fresh waters with a mineralization of up to 390 mg l^−1^ are locally distributed in the Filippovka riv. At the same time, the Zhuravlikha, Bystrukha, Khariuzovka, and Gromotukha rivers have ultra-fresh waters with a mineralization of less than 100 mg l^−1^.

In terms of hardness, the Zhuravlikha, Malaya Zhuravlikha, Breksa, Khariuzovka, Gromotukha, Tikhaya, and Ulba rivers have “soft” waters, while the Filippovka and Bystrukha rivers have to “hard” water. These waters are not in agreement with the Hygienic Standards established by the Republic of Kazakhstan,^34^ in terms of the hardness of the Filippovka riv. and Bystrukha riv.

**Fig. 2:**
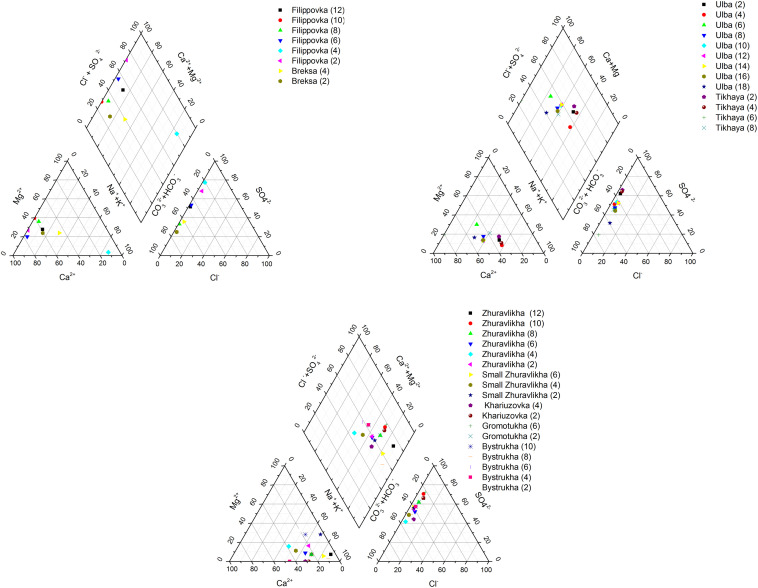
Piper diagram of the chemical composition of the waters of the small rivers of the Ulba river basin. The number of sampling points are in brackets.

The chemical composition of the surface waters of the small rivers of the Ulba r. basin is presented in the Piper diagram in Fig. 2. This diagram mainly consists of two triangular fields, each representing the composition of cations and anions, and a diamond-shaped field, representing the composition of cations and anions present in waters, and it allows a more detailed classification of waters using their major cations and anions.^37^

**Fig. 3:**
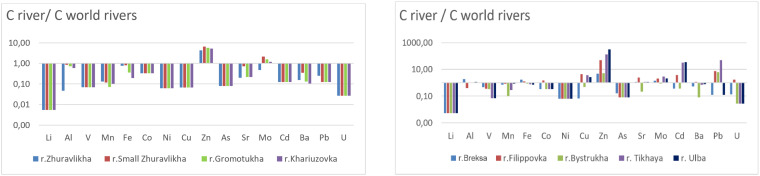
Comparison of the average concentrations of microelements in small rivers (a) Zhuravlikha riv., Malaya Zhuravlikha riv., Gromotukha riv., and Khariuzovka riv. as well as (b) Breksa riv., Filippovka riv., Bystrukha riv., Tikhaya riv., and Ulba riv., estimated in this study to the corresponding average concentrations in the rivers of the world.^38^

According to the Piper diagram (Fig. 3), the predominant chemical type of waters in the Zhuravlikha riv., Malaya Zhuravlikha riv., Khariuzovka riv., Gromotukha riv., and Bystrukha riv. is sodium–potassium sulphate. It was revealed that the waters of the Breksa riv. belong to hydrocarbonate calcium–magnesium. In the upper reach of the Filippovka riv. (p.12, p.10, p.8 and p.6), the waters are hydrocarbonate calcium–magnesium and mixed-type sulphate-hydrocarbonate calcium–magnesium. In the lower reach of the Filippovka riv., the waters at p.4 and p.2 belong to sodium–potassium sulphate and sulphate–calcium, respectively.

In the upper reaches of the Ulba riv., the waters at p.2 and p.4 belong to sodium–potassium sulphate, and most of the samples (p.6, p.8, p.10, p.12, p.14, and p.16) belong to the zone of mixed chemical type waters, namely, hydrocarbonate–sulphate sodium–calcium. In the lower reaches of the Ulba riv. (p.18), waters belong to calcium–magnesium hydrocarbonate.

Similarly, the waters in the Tikhaya riv. in the upper reaches (p.2 and p.4) have a sulphate sodium–potassium composition, which transitions in the lower reaches to calcium hydrocarbonate waters (p.6) and waters of mixed chemical compositions (p.8), namely, hydrocarbonate–sulphate calcium–sodium–potassium waters.

### 3.2 Micro elemental composition of the small rivers of the Ulba riv

The elemental compositions of the waters of the studied rivers are presented in Table 2.

**Table 2:** Contents of chemical elements in the waters of the small rivers of the Ulba riv. basin, μg L^−1^^a^

River name	Li	Al	V	Mn	Fe	Co	Ni	Cu	Zn	As	Sr	Mo	Cd	Ba	Pb	U
Zhuravlikha riv. (*n* = 13)	<0.02	**1.5**	<0.1	**4.5**	**50**	<0.1	<0.1	<0.2	**2.6**	<0.1	**12**	**0.20**	<0.02	**3.6**	**0.02**	<0.02
		*1.5–32*		*2.1–7.0*	*28–200*				*1.4–25*		*0.05–45*	*0.03–0.90*		*1.5–10*	*0.02–6.5*	
		168%		36%	71%				145%		83%			53%	327%	
Malaya Zhuravlikha riv. (*n* = 7)	<0.02	**27**	<0.1	**4.0**	**53**	<0.1	<0.1	**0.1**	**3.9**	<0.1	**43**	**0.90**	<0.02	**8.0**	<0.02	<0.02
		*1.5–68*		*1.2–14*	*19–240*			*0.1–1.1*	*0.6–8.0*		27–50	*0.83–0.92*		*5.0–26*		
		75%		85%	103%			124%	60%		23%	3.2%		71%		
Breksa riv. (*n* = 5)	<0.02	**59**	**0.33**	**25**	**110**	**0.05**	<0.1	<0.2	**2.9**	**0.1**	**67**	**0.60**	<0.02	**12**	**0.01**	**0.05**
		*24–320*	*0.24–1.13*	*2.3–320*	*21–750*	*0.05–0.35*			*2.2–4.4*	*0.1–2.0*	*56–96*	*0.14–1.0*		*5.4–19*	*0.01–2.6*	*0.05–20*
		109%	72%	177%	109%	102%			29%	177%	25%	56%		48%	219%	217%
Filippovka riv. (*n* = 11)	0.01	**13**	**0.25**	**28**	**83**	**0.22**	**0.05**	**6.5**	**30**	**0.05**	**148**	**0.84**	**0.30**	**27**	**0.60**	**0.64**
	*0.01–28*	*1.5–110*	*0.05–0.56*	*4.7–210*	*16–120*	*0.05–2.15*	*0.05–4.2*	*1–40*	*5.3–400*	*0.05–1.4*	*13–454*	*0.50–16*	*0.13–3.0*	*3.0–34*	*0.01–4.2*	*0.01–4.2*
	330%	154%	70%	104%	59%	142%	175%	116%	149%	127%	66%	171%	138%	42%	144%	144%
Bystrukha riv. (*n* = 11)	<0.02	**32**	**0.24**	**3.4**	**56**	<0.1	0.05	**0.70**	**3.1**	<0.1	**13**	**0.40**	<0.02	**1.8**	**0.5**	<0.02
		*21–75*	*0.05–0.42*	*1.7–91*	*41–410*		*0.05–3.9*	*0.10–1.3*	*1.5–10*		*11–20*	*0.04–0.70*		*0.05–2.60*	*0.01–0.9*	
		50%	73%	187%	103%		290%	53%	71%		22%	42%		56%	66%	
Khariuzovka riv. (*n* = 4)	<0.02	**19**	**<0.1**	**3.5**	**13**	<0.1	<0.1	<0.2	**3.1**	<0.1	**13**	**0.50**	<0.02	**2.4**	<0.02	<0.02
		*5.0–27*	*<0.1–0.12*	*1.0–4.6*	*10–17*				*1.2–4.7*		*13–14*	*0.50–0.56*		*2.3–2.6*		
		59%		52%	27%				49%		4%	6%		6%		
Gromotukha riv. (*n* = 5)	<0.02	**23**	<0.1	**2.4**	**24**	<0.1	<0.1	**0.1**	**3.4**	<0.1	**13**	**0.66**	<0.02	**3.0**	**0.01**	<0.02
		*1.5–37*		*0.5–11*	11–39			*0.1–4.5*	*3.0–7.3*		12–90	*0.54–1.20*		*2.0–8.0*	*0.01–1.5*	
		88%		115%	51%			20%	44%		124%	35%		66%	141%	
Tikhaya riv. (*n* = 9)	<0.02	**32**	<0.1	**9.4**	**49**	<0.1	<0.1	**5.5**	**80**	<0.1	**66**	**1.2**	**2.5**	**16**	**3.9**	**0.01**
		*1.5–51*		*1.0–21*	*10–72*			*0.1–7.0*	*20–190*		*44–87*	*0.80–1.5*	*0.1–5.0*	*7.0–19.0*	*0.01–8.9*	*0.01–0.36*
		60%		69%	50%			62%	59%		23%	18%	53%	30%	76%	195%
Ulba riv. (*n* = 20)	<0.02	**37**	<0.1	**29**	**47**	**0.05**	<0.1	**4.0**	**185**	<0.1	**63**	**0.90**	**2.7**	**18**	**0.01**	**0.01**
		*1.5–110*		*1.0–320*	*18–310*	*0.05–1.7*		*0.1–38*	*8.0–2800*		*16–130*	*0.17–1.00*	*0.03–21*	*3.0–68*	*0.01–6.80*	*0.01–0.10*
		70%		144%	113%	165%		153%	194%		40%	32%	146%	66%	136%	139%
Average content in the river waters of the world^38^	1.84	32	0.71	34	66	0.15	0.80	1.48	0.60	0.62	60	0.42	0.08	23	0.079	0.37
MPC for water^34^	30	500	100	100	300	100	100	1000	5000	50	7000	250	1	100	30	—
WHO MPC^35^	—	—	—	80	—	—	70	2000	—	10	—	70	3	1300	10	30
US EPA^39^	—	50–200	—	50	300	—	—	1300	5000	10	—	—	5	2000	—	—

a Bold–median, italic–min–max, Cv in brackets, %-variation coefficient.

The coefficients of variation for most of the studied elements are greater than 100%, which indicates a strong variability and data spread. Due to the presence of “outliers” in the sample, median values are taken as average values.

The cadmium content exceeded the MPC set by the Hygienic Standard of the Republic of Kazakhstan^34^ in the waters of the Ulba riv. (up to 21 MPC), Tikhaya riv. (up to 5 MPC), and Filippovka riv. (up to 3 MPC) in 65%, 88%, and 18% water samples, respectively. In general, the median cadmium values along the riverbed of the Tikhaya riv. and Ulba riv. were 2.5 μg l^−1^ and 2.7 μg l^−1^, respectively, which are higher than the standard levels.^34^

An increased lithium content was detected in the water at p.4 of the Filippovka riv., the concentration of which was at the MPC level. Even though the median Mn value in the waters of the Filippovka riv. did not exceed the MPC, 36% of water samples had Mn values exceeding the permissible limits. In the water from Breksa riv., high concentrations of iron and manganese were recorded at p.3 up to 3.2 MPC and 2.5 MPC, respectively.

Compared with the WHO MPC,^35^ exceedances were recorded for manganese in the waters of the Ulba and Breksa rivers (up to 4 MPC), Bystrukha river (up to 1.1 MPC), and Filippovka river (up to 2.6 MPC). For cadmium, exceedances were observed in the waters of the Filippovka river (up to 1 MPC), Tikhaya river (up to 1.6 MPC), and Ulba river (up to 7 MPC).

Exceedances relative to the US EPA^39^ standards for drinking water were observed for aluminium in the waters of the Breksa river (up to 1.6 MPC) and for manganese in the waters of the Ulba and Breksa rivers (up to 6.4 MPC), Bystrukha river (up to 1.1 MPC), and Filippovka river (up to 2.6 MPC). For iron, exceedances were recorded in the waters of the Ulba river (up to 1 MPC), Bystrukha river (up to 1.4 MPC), and Breksa river (up to 2.5 MPC). For cadmium, exceedances were observed in the waters of the Tikhaya and Ulba rivers (up to 1 MPC and 4.2 MPC, respectively).

The presence of chemical contamination from surface reservoirs in the Ridder c. is evidenced in numerous previous studies.^12,13,40^ For example, studies conducted in 2016 (ref. 12) recorded high concentrations of zinc in the Bystrukha riv. (4.4 MPC) and Khariuzovka riv. (1.8 MPC). The concentration of lead also exceeded the permissible limits in the water of these rivers and was at the level of 1 MPC. On the contrary, our data did not reveal an exceedance of zinc and lead concentrations from the permissible concentration limits in all studied rivers, and particularly the Bystrukha and Khariuzovka rivers.

In most cases, lower concentrations of lithium, aluminum, vanadium, manganese, iron, cobalt, nickel, arsenic, barium, and uranium were detected in the waters of small rivers compared to the global averages (Fig. 1 and 2).

In the waters of the Zhuravlikha, Malaya Zhuravlikha, Gromotukha, and Khariuzovka rivers, the cadmium and lead contents were also 8 times and 4–8 times lower than the global averages, respectively (Fig. 3). Because the waters of the studied watercourses predominantly undergo a slightly alkaline reaction, this does not contribute to the migration of many elements (including iron, manganese, and most of the associated oxides of microelements, primarily the sulphide group).

At the same time, the zinc content in the waters of all studied rivers was 4–308 times higher than the global average, with the waters of the Filippovka riv. (exceeding 50 times) and Tikhaya riv. maximally enriched with zinc.

The cadmium content in the waters of the Tikhaya riv. (31 times) and Ulba riv. (34 times) exceeded the global average. The lead concentration in the waters of the Filippovka riv. (8 times), Bystrukha riv. (6 times) and Tikhaya riv. (49 times) exceeded the global river average.

The high average concentration of Zn, Cd and Pb (more than 5 times) in the waters of the Filippovka, Tikhaya and Ulba rivers is due to the development of mineral deposits in the region and the influence of the mining and processing complex.

### 3.3 Assessment of the water quality in the small rivers of the Ulba riv. basin

**Fig. 4:**
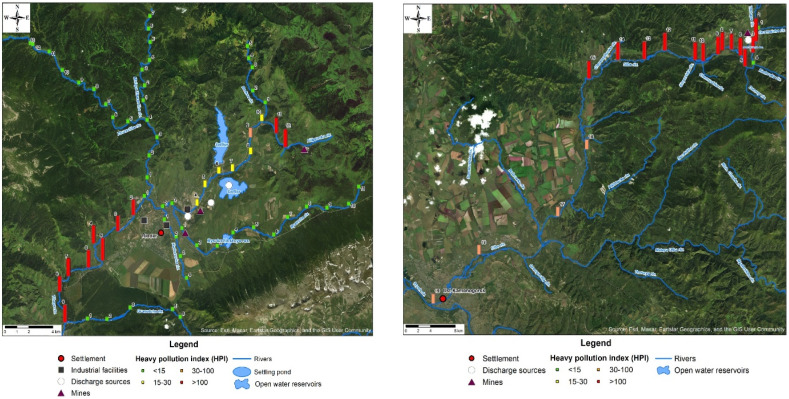
Values of the heavy metal pollution index for small rivers of the Ulba riv. basin.

To access the pollution degree and determine the suitability of water for household water use, the heavy metal pollution index (HPI) was calculated, and the results of the spatial distribution are shown in Fig. 4.

The waters of the Zhuravlikha, Malaya Zhuravlikha, Gromotukha, Khariuzovka, Bystrukha and Breksa rivers belong to the low level (<15), with no elevated heavy metal contents.

The results of the HPI in the identified polluted rivers are presented in Tables 3–5.

**Table 3:** Pollution condition of the Filippovka riv.

Sampling point	HPI	Chemical element exceeding MPC^34^
p.12	255	Mn, Cd
p.11	178	Cd
p.10	18	—
p.9	44	Mn
p.8	28	—
p.7	24	—
p.6	26	—
p.5	21	—
p.4	29	—
p.3	15	—
p.2	11	—
**Average**	**59**	—

**Table 4:** Pollution condition of the Tikhaya riv.

Sampling point	HPI	Chemical element exceeding MPC^34^
p.1–1	9	—
p.1	161	Cd
p.2	194	Cd
p.3	253	Cd
p.4	421	Cd
p.5	337	Cd
p.6	210	Cd
p.7	169	Cd
p.8	228	Cd
**Average = 220**		

**Table 5:** Pollution condition of the Ulba riv.

Sampling point	HPI	Chemical element exceeding MPC^34^
p.1	2.6	—
p.2	1769	Mn, Cd
p.3	194	Cd
p.4	8	—
p.5	228	Cd
p.6	228	Cd
p.7	177	Cd
p.8	303	Cd
p.9	295	Cd
p.10	303	Cd
p.11	270	Cd
p.12	253	Cd
p.13	244	Cd
p.14	253	Cd
p.15	227	Cd
p.16	51	—
p.17	51	—
p.18	76	—
p.19	76	—
**Average = 264**		

The waters of the Filippovka riv. have a high level of pollution (>100) in the upper reaches (p.12 and p.11), a middle level of pollution (15–100) in the downstream and a low level of pollution (<15) at the mouth.

The waters of the Tikhaya riv. are highly polluted (>100) and are characterized by elevated cadmium levels. The exception is p.1, which is located at the confluence of the Filippovka riv. and Tikhaya riv.

According to HPI indicators, the waters in the upper reaches of the Ulba riv. at most of the studied points are classified as highly polluted (>100) and are classified as mildly polluted only at the mouth (at points 16–19). The main pollutants in the Ulba riv., with concentrations exceeding the MPC, are cadmium and manganese (at one point). It should be highlighted that at p.2 in the Ulba riv., critical water pollution with heavy metals was detected (HPI = 1716). It should also be noted that even though the discharge of wastewater into the Ulba riv. happens in the upper reaches of the river (in the areas of p.2 and p.7), the river was also polluted downstream, namely, p.15 (high level of pollution) and p.19 (middle level of pollution).

On the basis of the average values of the water pollution index (HPI) along the riverbed, the following order of pollution levels can be established: River Ulba (264) > River Tikhaya (220) > River Filippovka (59). High and middle levels of water pollution were recorded near industrial plants, mines, and near wastewater^25^ discharges located within the catchment areas of the Filippovka riv., Tikhaya riv. and Ulba riv.

The high concentrations of cadmium in the waters, as indicated by the HPI, may have potential health consequences for the local population due to long-term exposure exceeding the MPC.^39,41,42^ These consequences could include kidney damage and an increased risk of cancer.

The undeniable impact of mining activities in the region is corroborated by historical data^43^ and numerous recent studies.^12,18,19,40^ For example, studies from 1959–1961 (ref. 43) indicated significant pollution of the Filippovka, Tikhaya, and Ulba rivers due to the discharge of industrial waste from the Leninogorsk Polymetallic Plant. According to the recent data from the annual reports of Kazhydromet,^18,19^ the Breksa, Tikhaya, and Ulba rivers are classified as watercourses with high and moderate levels of pollution, where copper, zinc, and cadmium pose the greatest environmental risks. Additionally, a known case of an emergency discharge occurred in 2016 from the Talovskoye tailings dump of the mining and processing plant in Ridder.^44,45^ This incident resulted in the release of decades-old pulp stocks into the Filippovka River, which subsequently spread to the Tikhaya and Ulba rivers.

Thus, the studied rivers are subject to the combined influence of both natural sources, because the region belongs to the Rudny Altai province (Ridder ore region), and anthropogenic sources, particularly the impact of mining operations.

In light of these factors, the long-term monitoring of the small rivers in the Ulba River basin of Eastern Kazakhstan is essential to assess water quality under varying levels of environmental stress.

## 4 Conclusions

The waters of the small rivers studied mainly are ultra-fresh, slightly alkaline. At the same time, the Zhuravlikha, Bystrukha, Khariuzovka, and Gromotukha rivers have ultra-fresh waters with a mineralization of less than 100 mg l^−1^. The chemical type of the waters studied is sodium–potassium sulphate, calcium–magnesium hydrocarbonate, and mixed chemical type, namely, hydrocarbonate–sulphate sodium–calcium.

The water samples do not meet the Hygienic Standards established by the Republic of Kazakhstan in terms of hardness in the Filippovka riv. and Bystrukha riv. The cadmium content exceeds the MPC set by the Hygienic Standards of the Republic of Kazakhstan in the waters of the Ulba riv. (up to 21 MPC), Tikhaya riv. (up to 5 MPC) and Filippovka riv. (up to 3 MPC) in 65%, 88% and 18% of water samples, respectively. For the Filippovka riv., 36% of water samples showed manganese values exceeding the MPC by 1.0–2.1 MPC, and in one sample, the concentration of lithium in the water was at the level of 1 MPC. For the Breksa riv., high concentrations of iron and manganese, recorded at p.3, were up to 3.2 MPC and 2.5 MPC, respectively.

The exceedances of manganese, aluminium, iron, and cadmium in the waters of the Ulba, Filippovka, Breksa, and Bystrukha rivers, based on the WHO MPC and US EPA standards, were recorded, ranging from 1 to 7 times. The highest exceedances were observed for manganese in the Ulba and Breksa rivers, with concentrations exceeding the WHO standards by 4 times and 6.4 times, respectively. For cadmium, the maximum exceedances were observed in the Ulba river (7 times the WHO standard) and Breksa river (4.2 times the US EPA standard).

In most cases, lower concentrations of lithium, aluminum, vanadium, manganese, iron, cobalt, nickel, arsenic, barium, and uranium were detected in the studied small rivers compared with the average contents in the river waters of the world. At the same time, high average concentrations of Zn, Cd, and Pb (more than 5 times the global average) were detected in the waters of the Filippovka, Tikhaya and Ulba rivers.

According to HPI indicators, high and middle levels of water pollution were recorded in the areas of wastewater discharge along the entire riverbed of the Tikhaya riv. and Ulba riv. and in the upper reaches of the Filippovka riv. The waters of these rivers are not suitable for drinking and cooking purposes. At the same time, water deterioration of these rivers can lead to the degradation of aquatic and coastal ecosystems. A low level of pollution (HPI < 15) was observed where there were no elevated concentrations of the studied heavy metals, including the waters of the following small rivers: Zhuravlikha, Malaya Zhuravlikha, Gromotukha, Khariuzovka, Bystrukha and Breksa.

Thus, it can be concluded that the increased concentrations of detected heavy metals in the Filippovka, Tikhaya and Ulba rivers are associated with geological sources and the long-term man-made influence of mining enterprises in the region.

To mitigate significant risks to the integrity of regional water resources and human health, it is recommended to enhance the regulation and management of water resources in the extractive industry. Specifically, this should include the adoption of advanced technologies for industrial water treatment and the implementation of measures to prevent emergency situations.

## Abbreviations

MPC: Maximum permissible concentrations
GOST: Government standard
ICP-MS: Inductively coupled plasma mass spectrometry
ICP-AES: Inductively coupled plasma atomic emission spectroscopy
HPI: Heavy metal pollution index
WHO: World Health Organization

## Data availability

All figures and tables quoted in this study were created by the authors.

## Author contributions

Madina Dyusembayeva: methodology, formal analysis, investigation, visualization, writing – original draft, writing – review & editing. Azhar Tashekova: conceptualization, validation, writing – review & editing, project administration. Yerbol Shakenov: methodology, formal analysis. Vladimir Kolbin: formal analysis, investigation. Nazgul Nurgaisinova: formal analysis, investigation. Ainur Mamyrbayeva: formal analysis, investigation, validation. Marija Abisheva: formal analysis, visualization, investigation.

## Conflicts of interest

There are no conflicts to declare.
